# An Evolutionary Framework for Association Testing in Resequencing Studies

**DOI:** 10.1371/journal.pgen.1001202

**Published:** 2010-11-11

**Authors:** C. Ryan King, Paul J. Rathouz, Dan L. Nicolae

**Affiliations:** 1 Department of Health Studies, University of Chicago, Chicago, Illinois, United States of America; 2 Department of Biostatistics and Medical Informatics, University of Wisconsin-Madison, Madison, Wisconsin, United States of America; 3 Departments of Medicine, Statistics, and Human Genetics, University of Chicago, Chicago, Illinois, United States of America; University of Oxford, United Kingdom

## Abstract

Sequencing technologies are becoming cheap enough to apply to large numbers of study participants and promise to provide new insights into human phenotypes by bringing to light rare and previously unknown genetic variants. We develop a new framework for the analysis of sequence data that incorporates all of the major features of previously proposed approaches, including those focused on allele counts and allele burden, but is both more general and more powerful. We harness population genetic theory to provide prior information on effect sizes and to create a pooling strategy for information from rare variants. Our method, EMMPAT (Evolutionary Mixed Model for Pooled Association Testing), generates a single test per gene (substantially reducing multiple testing concerns), facilitates graphical summaries, and improves the interpretation of results by allowing calculation of attributable variance. Simulations show that, relative to previously used approaches, our method increases the power to detect genes that affect phenotype when natural selection has kept alleles with large effect sizes rare. We demonstrate our approach on a population-based re-sequencing study of association between serum triglycerides and variation in ANGPTL4.

## Author Summary

Studies correlating genetic variation to disease and other human traits have examined mostly common mutations, partly because of technological restrictions. However, recent advances have resulted in dramatically declining costs of obtaining genomic sequence data, which provides the opportunity to detect rare genetic variation. Existing methods of analysis designed for an earlier era of technology are not optimal for discovering links to rare mutations. We take advantage of 1) the advanced theoretical understanding of evolutionary mechanics and 2) genome-wide evidence about evolutionary forces on the human genome to suggest a framework for understanding observed correlations between rare genetic variation and modern traits. The model leads to a powerful test for genetic association and to an improved interpretation of results. We demonstrate the new method on previously confirmed results in a gene related to high blood cholesterol levels.

## Introduction

Over the past 20 years, positional cloning guided by linkage analysis and genome wide association studies (GWAS) have identified many loci relevant to human disease and other quantitative phenotypes such as height, body mass index, and serum lipid composition. However, in most cases the total amount of phenotypic variance explained is small compared to the heritability observed in twin or adoption studies [1]. Some authors note the possibility that low-frequency genetic variation, which is not measured on standard single nucleotide polymorphism (SNP) arrays, may contribute to this missing heritability [2]–[7]. The rapidly decreasing cost of obtaining DNA sequence has prompted several groups to test this hypothesis by sequencing candidate genes in participants of cohort or case-control studies hoping to discover either 1) rare or previously unknown SNPs with large detectable effect sizes, or 2) a correlation between overall number of rare SNPs and phenotype [8]–[15]. This research is rapidly approaching a new phase as investigators use next-generation sequencing technology to measure all variation in the exome and wider genome [16], [17]. Several authors have shown that rare variation is particularly relevant in the case that natural selection has acted to keep variants with large effects rare, and that without action by purifying selection rare variants have effect sizes comparable to common ones [2], [3], [6].

There are three signatures of association in a resequencing study which we want to use to assess candidate genes. Some SNPs could have effect sizes large enough that they have individually noticeable impact on phenotype; this is the information underlying regression procedures, like those put forward by Hoggart et al [18] and Kwee et al [19]. This approach is very similar to current tag-SNP based procedures and not designed thinking of resequencing data, since the effects of rare SNPs will not be easy to discern. Depending on the role natural selection has played in the history of the phenotype, two other signatures of association may exist. Second, rare SNPs may tend to have effect sizes in the same direction (e.g. inducing risk), so a measure of overall rare-variant burden could correlate to phenotype; this is the information exploited in allele-count [20] and rare-variant-burden [21] type methods. That signature may be present if either selection has favored the phenotype (or a correlate) in a particular direction, or if purifying selection has been weak and derived alleles tend to be deleterious to the phenotype. Finally, rare SNPs could tend to have effect sizes which are larger than common ones. This could be the case if selection has tended to stabilize the phenotype. The method of Kwee et al [19] can allow for that possibility, but does not contain guidance on what the structure of the frequency - effect size relationship should be.

We present a method capable of detecting all three signatures of association. Our method generalizes allele count and rare-variant-burden methods by explicitly constructing a model relating disease impact, selective pressure, and SNP frequency in a candidate gene. By doing so, we will be able to provide intuitive interpretations to detected associations, allowing investigators to answer additional questions with their data. Our approach will yield substantially more power if the model is close to correct without introducing bias or sacrificing much efficiency when our assumptions are not met.

We propose to estimate the evolutionary fitness burden of each SNP using its observed frequency and population genetic parameters inferred by other authors. That estimate of fitness burden will act as prior information on the variant effect, acting like a burden function [21]. The same estimate will structure the variability of SNP-phenotype correlations, replacing arbitrary weights [19], and provide robust estimates even if there is no relationship between fitness and effect magnitude. We recognize that for a quantitative trait measured in a prospective cohort, a well-justified approximation of the full model can be fit using a fast and general statistical technique, mixed linear models, and provide software routines to estimate parameters and conduct hypothesis tests. We have named the approach EMMPAT (*E*volutionary *M*ixed *M*odel for *P*ooled *A*ssocation *T*esting)

In what follows, we will briefly introduce the population genetics ideas which underly our approach. Next, we construct our statistical model and discuss estimation and testing within it. Finally, we illustrate the method both in simulation studies and on a real candidate gene resequencing study examining serum triglyceride levels in a multi-ethnic prospective community-based sample [8], [12].

### Relating SNP Frequency, Fitness, and Disease Effect

Several authors have reviewed the potential contribution of low frequency alleles to variation in phenotypes [2]–[7]. Absent a change in the properties of new mutations during recent history, which we find implausible, systematic differences between SNPs of varying frequencies must be mediated by natural selection. Since the early 20th century, much work has explicated the evolutionary dynamics of quantitative traits, reviewed by Barton and Johnson [22], [23]. Below we will posit a model of pleiotropic selection whereby the trait under study or a trait with a correlated genetic basis is under purifying selection. More detailed connection and contrast to the existing work on the genetic basis of quantitative traits is found in Text S1.

**Figure 1:**
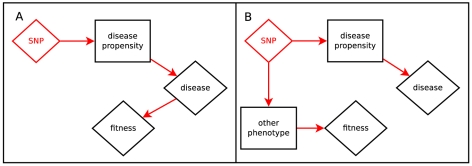
Hypotheses relating SNP effect and fitness effect. Panel A depicts the scenario where the trait is directly under selection. Panel B depicts the scenario where a gene with pleiotropic effects creates fitness-trait correlation via a related phenotype.

In Figure 1, we illustrate direct and apparent selection scenarios which give rise to a correlation between fitness effects and phenotype effects. In Figure 1A, the phenotype itself is under selective pressure; for example, disease leading to propensity to childhood mortality. Figure 1B shows apparent selection by pleiotropy; variants which disrupt an unconstrained role of a gene also tend to disrupt another role which is under selection; for example, variation which increases Alzheimer's Disease risk after reproductive age may relate to other brain function which is relevant for individuals still reproducing.

Hartl and Clark [24] carefully constructs and interprets the concept of fitness-effects in classical population genetics. Briefly, in an idealized population, the relative reproductive advantage of an individual is the product of the fitness effects of each variant that person carries, an additive approximation with no dominance or epistasis. We parameterize the problem in terms of the log of multiplicative fitness effects. That is, the fitness of the 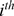 person is given by 
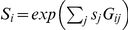
 where the fitness effect of the 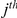 variant is denoted 
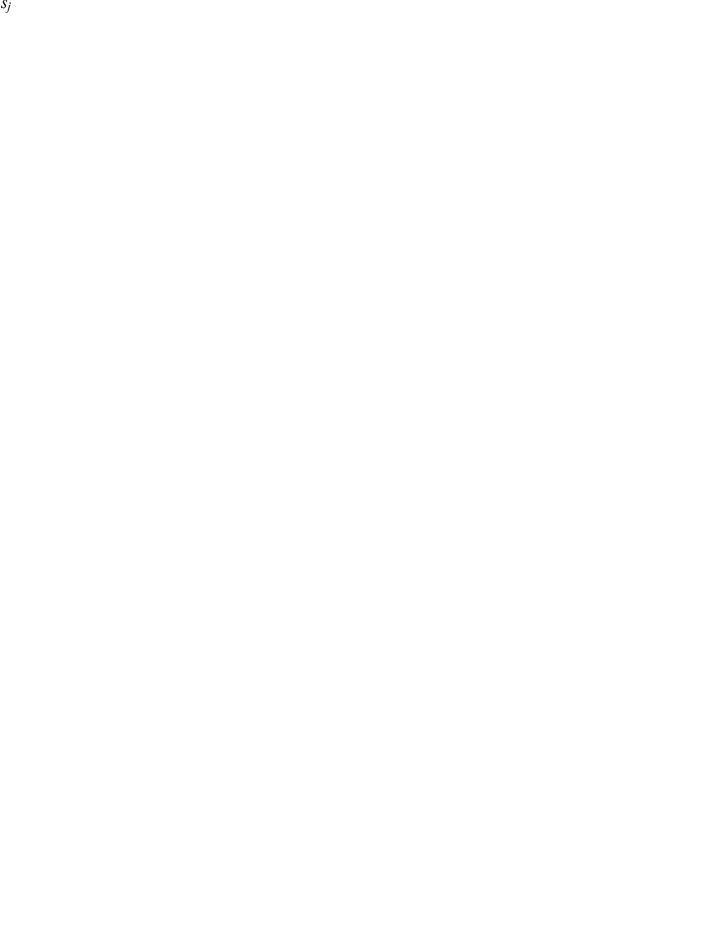
 and 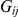 is the unphased genotype at that locus. The fitness effect of a new mutation 
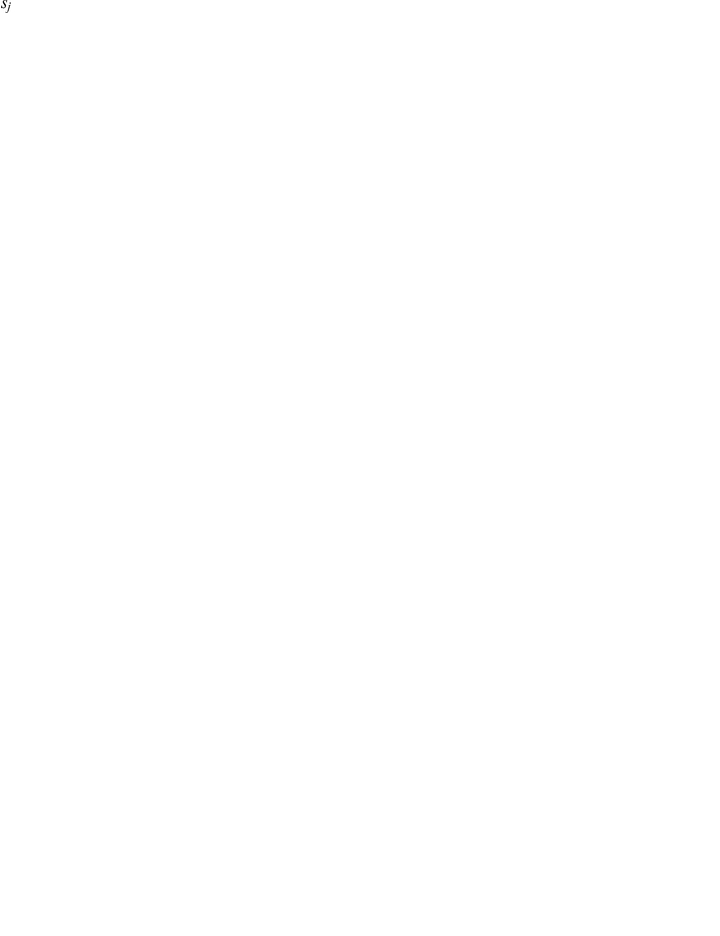
 determines several of its properties, such as average sojourn time before either going extinct or fixing at 100% prevalence and average frequency when sampled at a point in time [24].

Rather than assume that all variants in the region have the same 
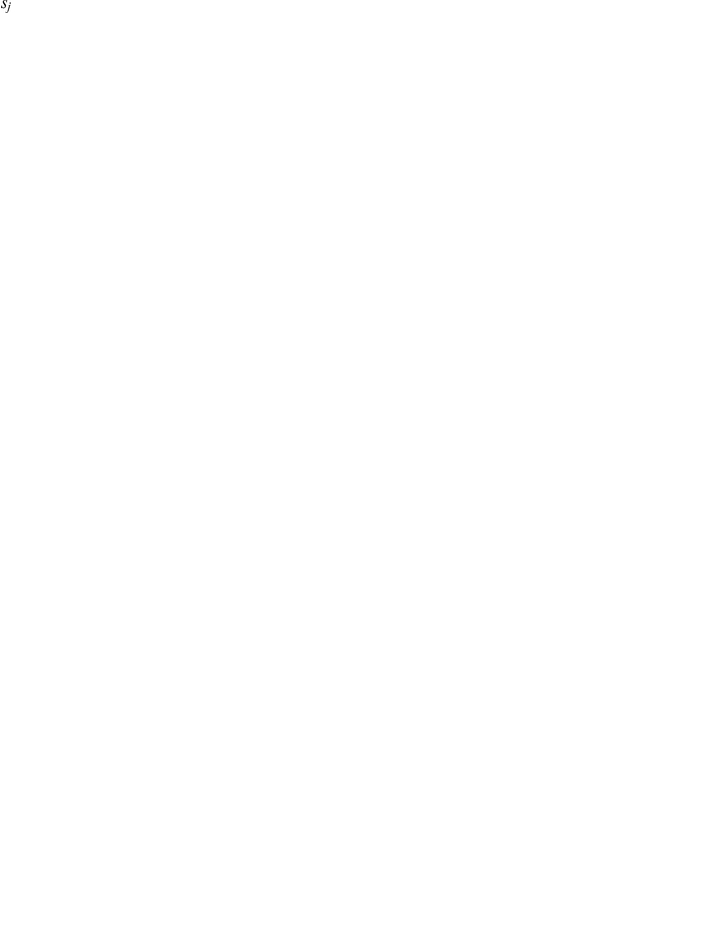
, we assume that the 
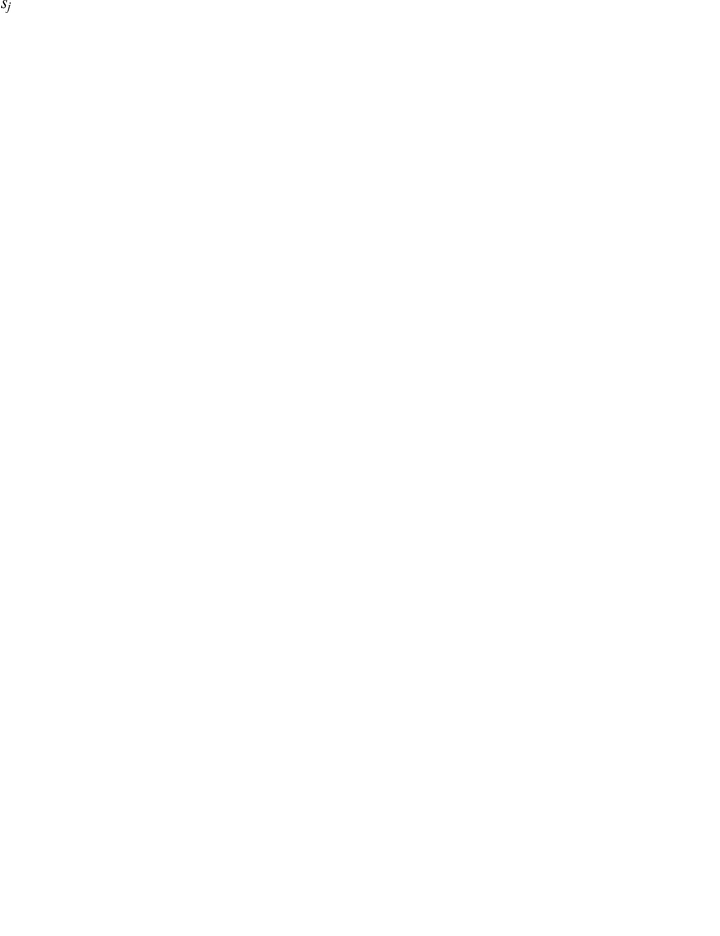
 of new mutations are sampled from a *distribution of fitness effects* (DFE). Just as a fixed 
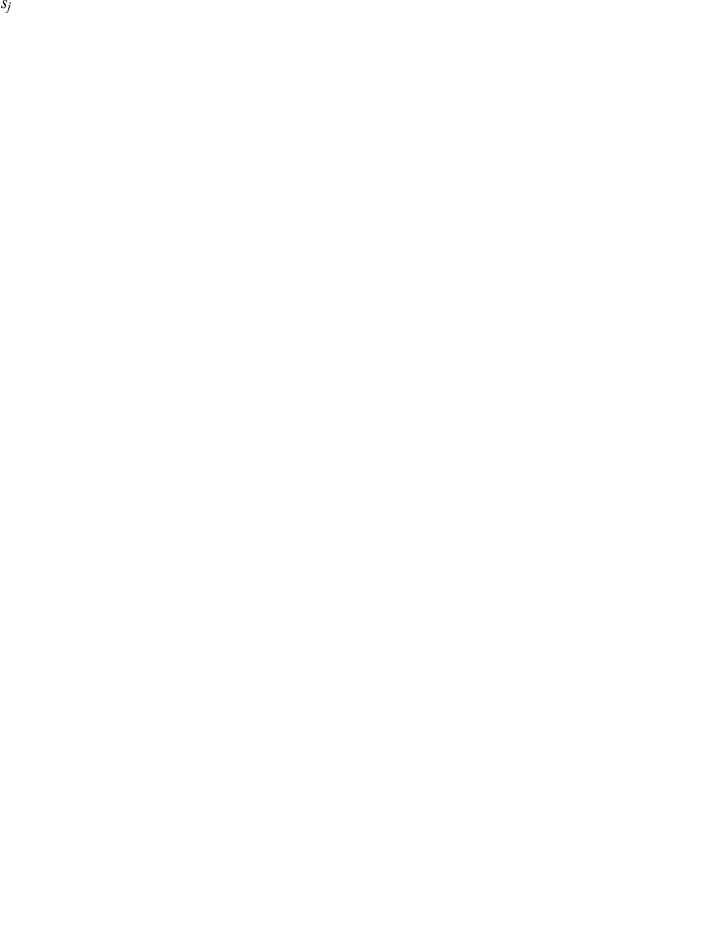
 would determine properties of the sampled genotype data for a SNP, a DFE along with mutation, recombination, and demographic parameters induces a distribution on the observed frequency spectrum and polymorphism - divergence ratios in sampled data. Several authors have attempted to fit a parameterized DFE from genomic data [25]–[34]. Boyko et al [33] found that a combination of a point mass at neutrality (not under selection) combined with a gamma distribution for deleterious differences from neutrality to be a good fit for the DFE of non-synonymous mutations.

With these facts in mind, in what follows we will use fitness effects to operationalize the construct of functional status for each SNP. Whereas Johnson and Barton [23] worked directly with the joint distribution of fitness and phenotype effects, we will use an existing DFE estimate [33] as a marginal distribution for fitness effects and construct the conditional distribution of phenotype effects. Since we do not know the true fitness effects of SNPs, we will estimate them with observed SNP frequency, which is statistically ancillary to phenotype-SNP correlation, using a simulation methodology described below.

## Methods

### Model for SNP Effects on Phenotype

Assume the context of a simple random cross-sectional sample of 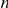 individuals (indexed by 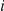) studying a quantitative trait 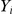 measured once per individual. Assume that these individuals also possess vectors of covariates 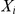 and genotypes 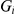 at each locus inside a sequenced candidate gene or region. The genotypes are coded such that “0” represents homozygous possession of the ancestral allele, “1” heterozygosity, and “2” homozygous possession of the derived allele at the locus. That is, 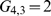 represents the fourth sampled person possessing two derived alleles at the third locus in the sequenced region.

We can write a regression model for person 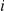
's phenotype 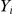 in terms of deviation 
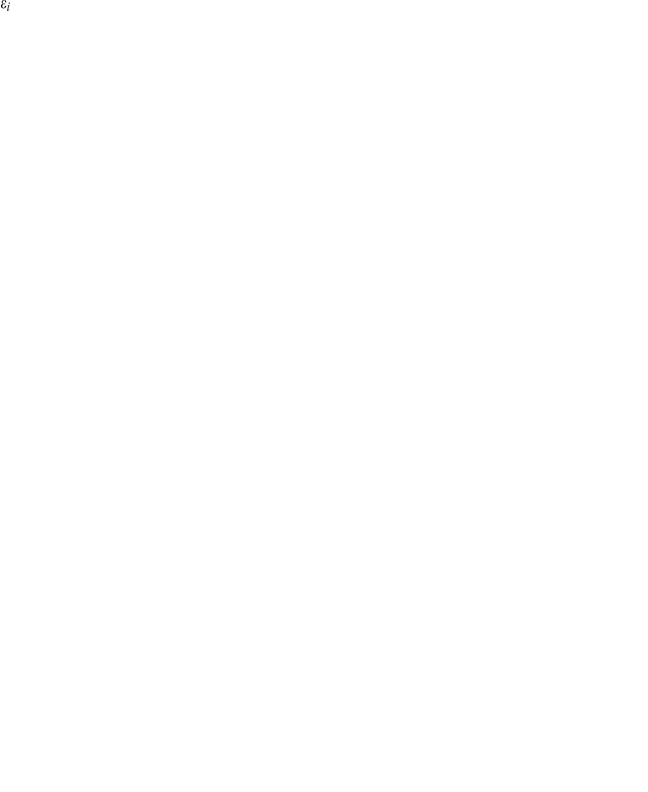
 from an average level predicted by covariate effects 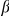 and additive genotype effects 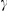,
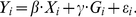
(1)


Using standard least-squares regression to estimate such a model will pose several problems. First, because there will be many rare variants, 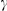 will contain many poorly estimated coefficients. The large number of rare variants will give model (1) a large number of degrees of freedom, decreasing its power to detect association with the candidate gene. Some of the variation uncovered may be perfectly correlated in the sample, meaning that those coefficients are not separately estimable in least-squares regression. Additionally, as the amount of the genome sequenced becomes large, there will be more variants than participants, making the entire model unidentified.

To overcome these problems, we need to make more assumptions and model the 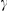 coefficients. We adopt a model where we view the effects of SNPs in the study as a sample from a wider population of SNP effects, and characterize that entire population using only three parameters. To fix ideas, assume for now that we knew the fitness effect of each SNP 
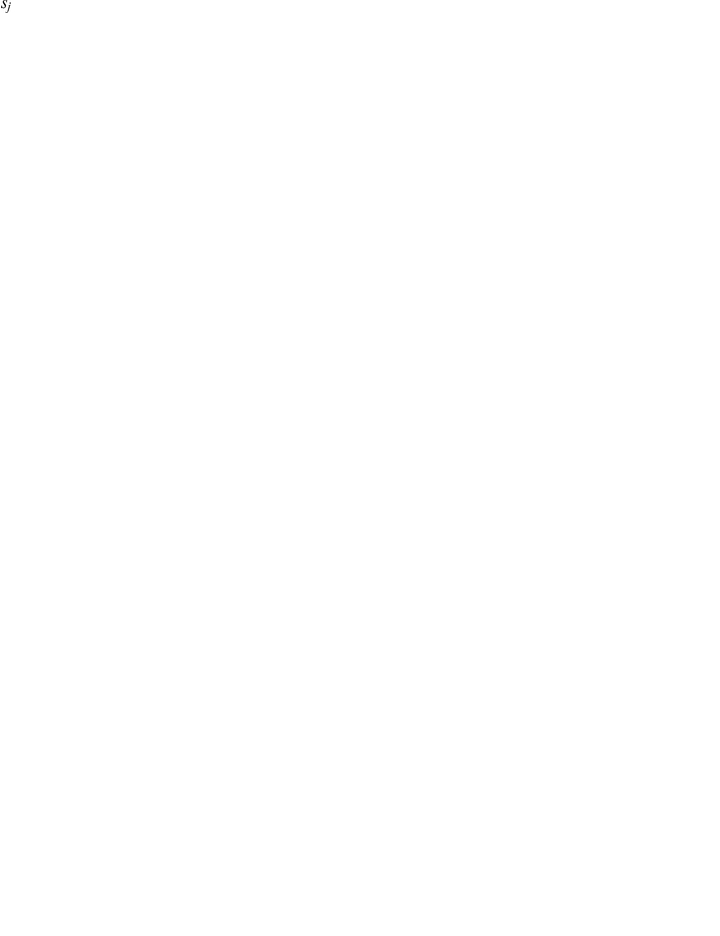
. If fitness was perfectly correlated to effect on phenotype, we would use that as a summary for all alleles, 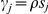, where the parameter 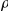 relates the scales of the two measures. As the fitness effect is not perfectly correlated to effect on phenotype, we add a mean 
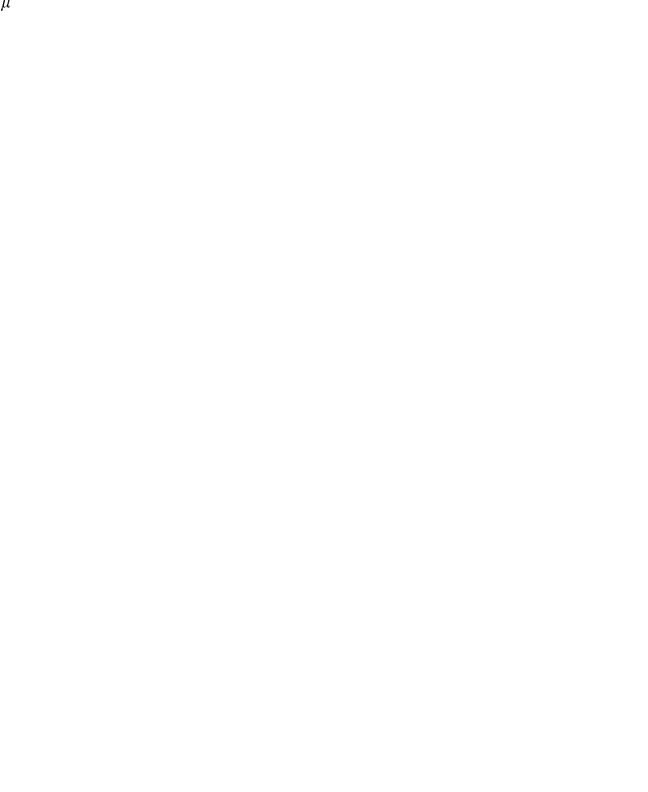
 and an error term 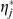 acknowledging those limitations to obtain
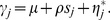
(2)In applied problems, 
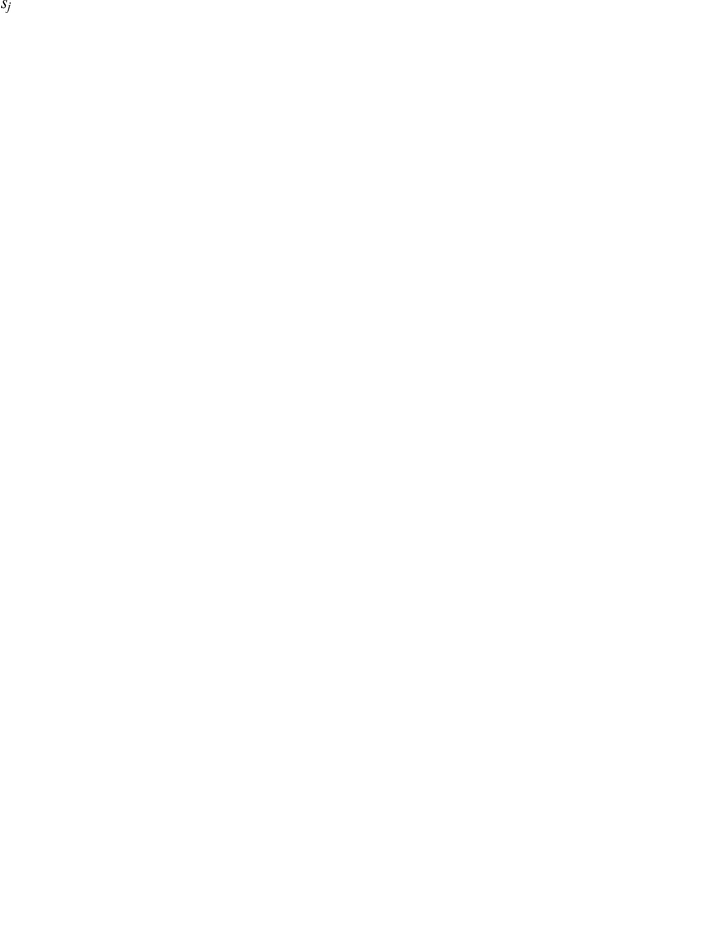
 is not known a-priori, so we will construct a prediction 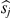 based on the observed frequency. We denote 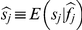 for that estimate, and for its prediction error we write 
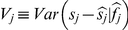
. We plug those estimates in to (2) to obtain
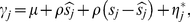
(3)and combine the two uncorrelated error terms to yield
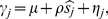
(4)where
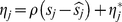
(5)

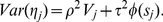
(6)The first term in (4), 
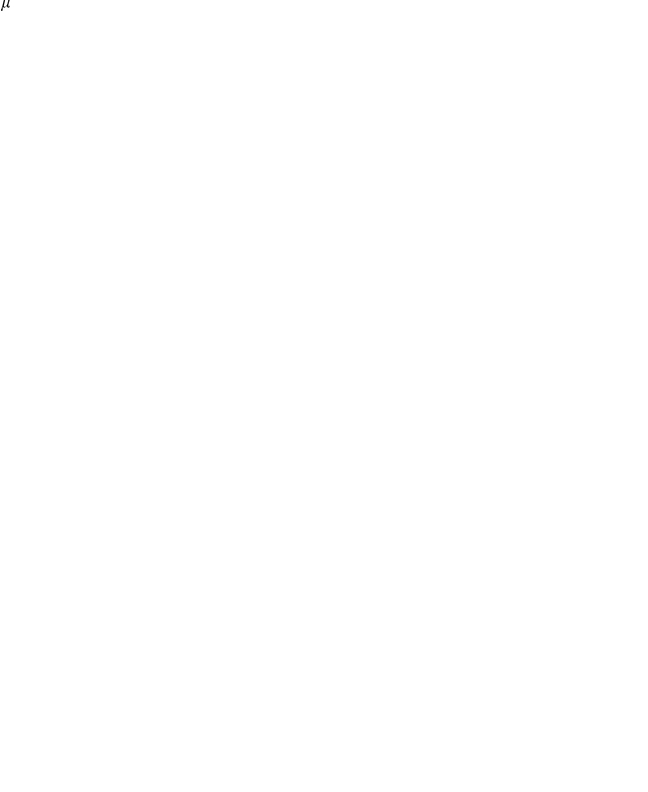
, allows derived alleles to on average increase or decrease the phenotype. The second term 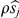 is an unscaled correlation between phenotype effects and expected fitness effects 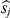. The error term 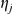 is the deviation in SNP *j*'s effect on phenotype from the average of SNPs with the same observed frequency. The variance of 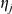 in (6) therefore has two components, first 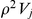 corresponds to prediction error of 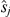, and second 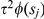 is the variance of phenotype effects for SNPs at the same level of true fitness burden 
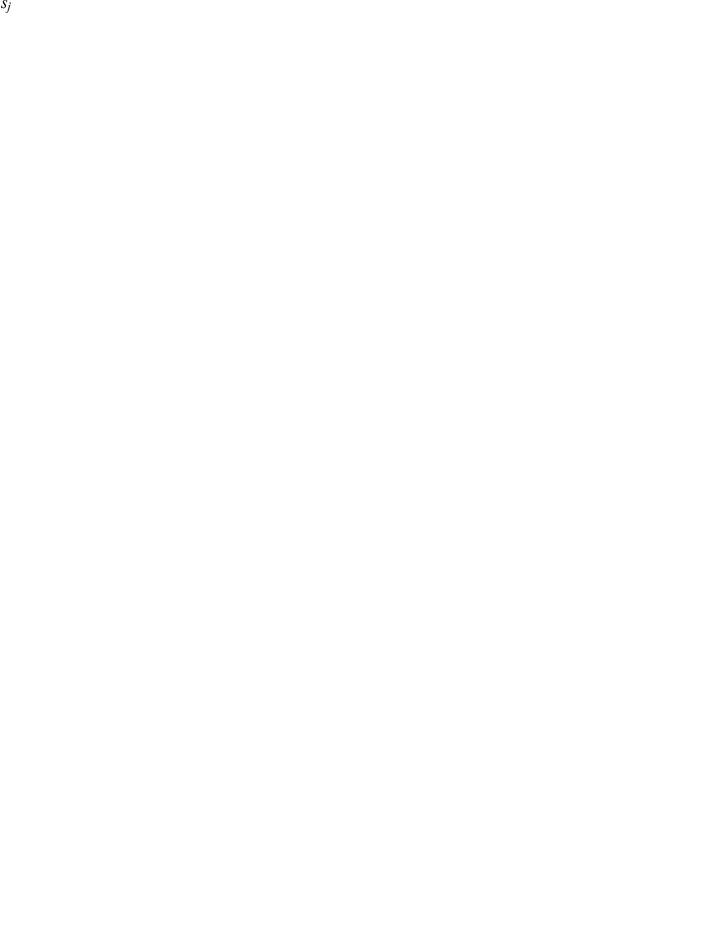
. The function 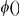 allows that as average burden changes the variability might also change. Although one could imagine “bad” alleles being more variable in their effects than relatively neutral alleles, implying non-constant 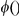, we propose constant 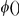 as a reasonable modeling start. This will still allow for the variance of effect sizes to change with observed frequency because of non-uniformity of 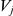 with frequency.

Equation (4) asserts that phenotype-effect and fitness-effect are linearly related; that seems correct for the scenario in Figure 1A and a good starting place for the other possibilities. In future work we will be able to empirically examine this assumption by graphical diagnostics and comparing fits using other functional forms. Further discussion of nonlinear relationships is found in Text S1, and we will demonstrate the impact of an incorrect assumption of linearity in our simulation studies.

Our model is quite general in that existing methods correspond to submodels of (4). An allele count method tests the model with only 
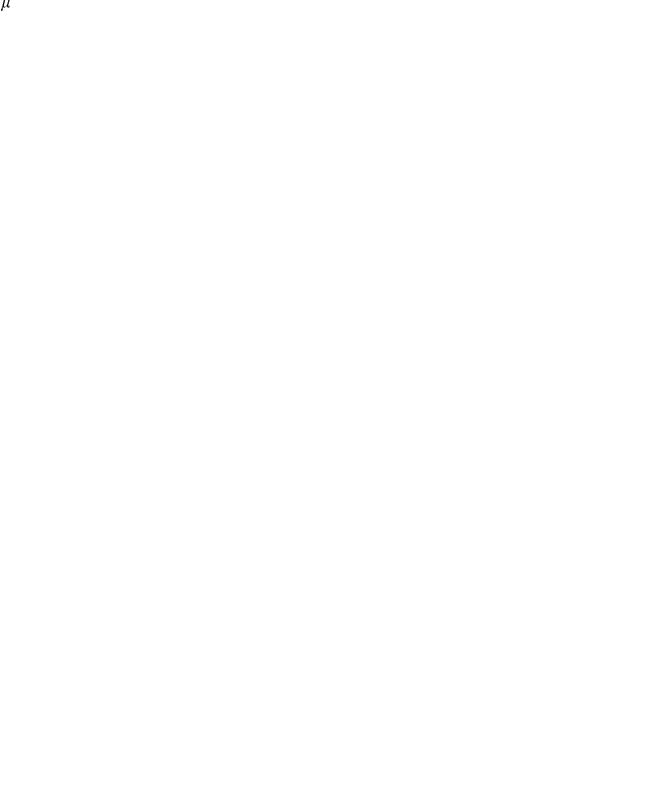
 allowed to vary; rare alleles below an arbitrary threshold are summarized by an average effect which does not change with frequency, so 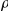 and all 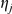 are set to zero, and alleles above that threshold are regarded as free parameters. Similarly a weighted-burden method corresponds to the model 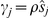 with a particular implementation of 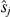, such as in Madsen et al [21] where 
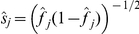
, and forces all 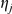 in the rare alleles to be zero. Our model will not involve an arbitrary threshold for “rare alleles” and will adaptively pool variant effects in a flexible way. As shown in the results, this will create substantial power gains in a variety of settings.

When 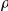 and 
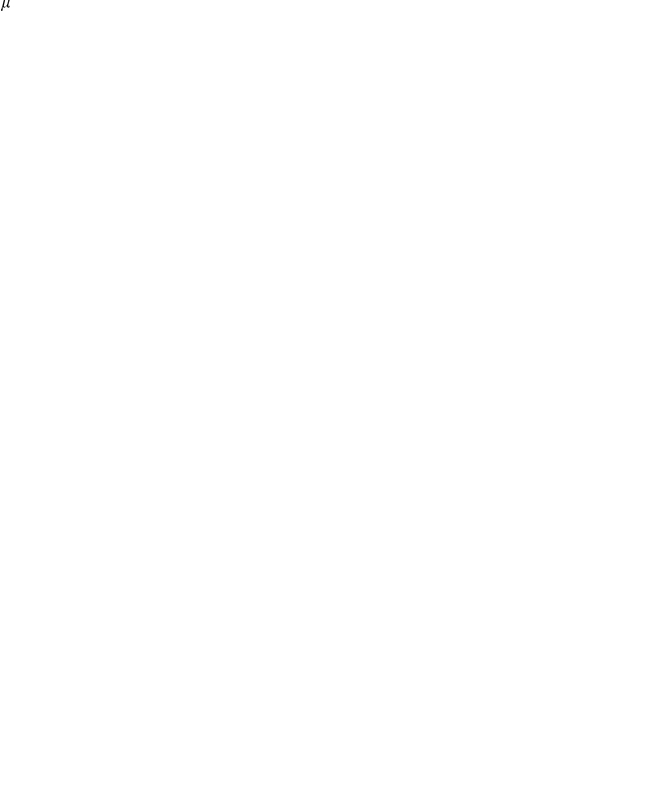
 in (4) are zero, our model reduces to a standard random-effect model identical to that of Kwee et al [19] with all variants given the same weight. That is, regardless of frequency all SNPs have the same likelihood of having large effect sizes, and regardless of frequency SNP effects have zero mean. As a result, our method will be robust to the case that fitness and phenotype effects are unrelated by estimating 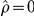 and retaining the flexibility of the method of Kwee et al. The major difference between the above and our method is the use of population genetics to suggest the structure of the variance of SNP effects, including a fallback should fitness and phenotype effect not be related. Kwee's method is developed in the context of tag SNPs and suggests an arbitrary variance of SNP effects given as either a constant, 
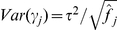
, or any prior-information based form. A related method is that of Hoggart et al [18]. Their approach corresponds to 
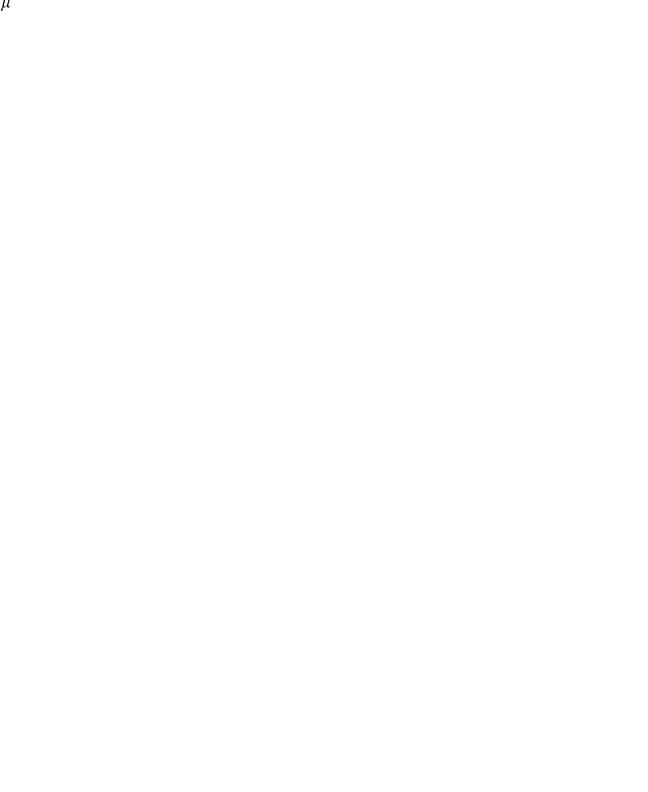
 and 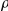 set to zero (they assume a mean-zero distribution) and a different set of restrictions on the distribution of 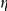. Their assumptions about the distribution of 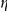 were chosen to yield estimates with most variants having zero effect, a feature called model selection which eliminates small effects and correlated variables. In contrast, our model will tend to reign in large effect sizes and split effect size between variants in high linkage disequilibrium, but does not eliminate SNPs from the fit. We prefer our choice for resequencing for several reasons. First, there may well be many effects of small size which are cumulatively important, and we want to retain those small effects in the model. Second, we want an estimate of the effect size of each variant for graphical and diagnostic purposes. Third, we accomplish a similar goal of reducing the model size by rejecting the null on a small number of genes. That is, we want to identify a small number of disease relevant genes with our efficient test; doing so will exclude most SNPs without further model selection procedures. Fourth, by smoothly grouping rare SNPs and summarizing them with only a few parameters, we already greatly reduce the multiple testing burden.

### Model Interpretation

**Figure 2:**
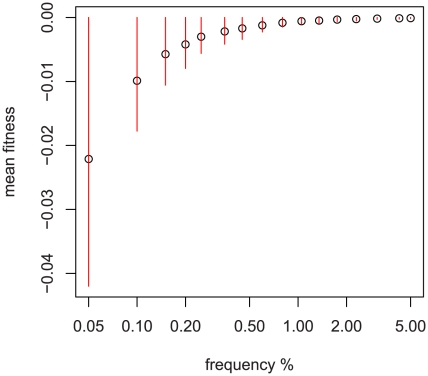
Relationship between sampled frequency and mean fitness. Simulation results using fitted DFE of non-synonymous variation from [33] and a sample size of 1000 diploids. Red bars are median +−35% of the distribution at that sampled frequency. The x-axis is logarithmic and scaled by 100, i.e., the first point is 1/2000 chromosomes.

The specification of equations (1), (4), and (6) yields a natural interpretation to the fitted model. After estimating the population parameters of phenotype effects, we will be able to jointly estimate individual SNP effects 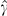 and their impact on the phenotype of each person in our sample. By calculating 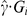, we obtain the expected difference between participant *i*'s phenotype and what we would expect were there no effects of this gene. As a result we can empirically estimate the overall phenotypic variability due to observed genetic variants, 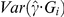 over study participants. We can similarly estimate the variability dues to rare alleles by including only rare SNPs in the above calculation. The overall effect 
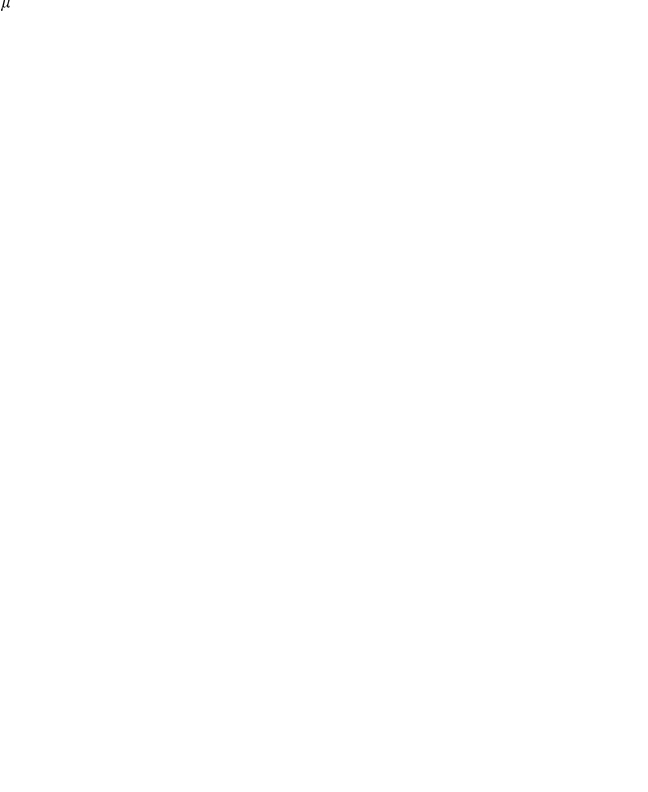
 is an average change in phenotype per derived allele, perhaps due to inadequate purifying selection. In the variance expression (6), 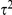 is the variability of allelic effects for a given level of true fitness. As will be shown below in Figure 2, when using the genome-wide distribution of fitness effects for non-synonymous SNPs, common variation is nearly neutral so 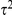 can also be thought of as the variability of effects of common alleles. 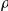 represents the correlation between fitness burden and phenotypic burden. This parameter's interpretation relies on accurately estimating the scale of fitness effects and has awkward units, but we can avoid this difficulty by noting that (4) can be decomposed into a fitness related portion and a fitness unrelated portion which are independent
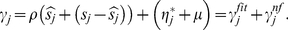
(7)By calculating 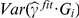 we can ascribe a proportion of total variation in phenotype to selection-phenotype correlation without worrying about having gotten the scale of 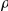 correct. Calculations for separating these variance components are found in Text S1. We can use the same technique to compare classes of SNPs, for example non-coding vs missense, by jointly fitting separate 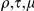 and comparing the attributable variance for each class of SNPs. We will illustrate this idea in our real data example. This decomposition also shows why it is not crucial for our estimates of fitness to be perfect. The model can fall back by setting 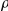 to zero and use only 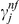 to recover a working model which does not pool information across rare alleles. Doing so will mean that the opportunity to gain information by recognizing structure in phenotype effects will not be realized, but the remaining estimation method is still valid.

An important consideration is how to interpret the results when multiple ethnic groups are analyzed simultaneously. Because some genetic variation is fixed between ethnic groups in the sample, the average effect of single-population variation will be absorbed into the fitted mean for that group. As a result, the interpretation for “total explained variation” is actually “total explained within-ethnic-group variation;” genetic variation may explain some of the phenotypic difference between groups, but we do not include it in our estimate because of confounding between environmental exposures and ethnic background.

Another point requiring clarification is the assumption that genotype effects are independent. In the context of GWAS, nearby SNPs often are thought to have correlated effects because they mutually tag a functional variant. Additionally, estimates of SNP effects will be correlated due to LD making their true separate effects difficult or impossible to identify. However, in the underlying data generating mechanism true genotype effects are independent. Because sequencing identifies all the variation within the region and eliminates much of the correlation due to untyped alleles, we believe that the independence assumption is a useful approximation in this case. Non-independence of the true effects could be accommodated by imposing a covariance structure on SNP effects, for example using their spatial distance in the genome or folded protein. Alternatively, the phylogenetic approach of TreeLD [35] estimates the degree of probable overlap of untyped SNPs.

### Computing Fitness Effects

Model (4) relies on a prediction 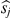 of the fitness effect of each variant as well as an estimate 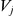 of the error of that prediction. We use the following procedure to calculate such estimates.

1. Take as given the fitted distributional form of fitness effects and population history since out-of-Africa [33], [36].
2. Use existing software SFS_CODE [36] to simulate new polymorphisms in the gene under study many times, creating pseudo-samples containing true variant-level fitness.
3. For each variant in the real dataset, find variants in the pseudo-data with the same sampled frequency, and calculate the mean 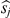 and variance 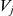 of true fitness among those simulated variants.

To reduce computational requirements, steps 2 and 3 above can be replaced by simulating a smaller number of large populations and calculating the expected mean and variance of fitness using simple random sampling. Figure 2 depicts the relationship of 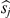 and 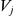 to frequency when using a genome-wide fitted DFE [33]. Because much of the variation discovered in our multi-ethnic example dataset is confined to one ethnicity, we use the ethnicity-specific frequency and pseudo-data. Because of admixture in our sample, we use the highest observed frequency (the most skeptical about its being rare) to assign an ethnicity of origin to SNPs appearing in multiple groups.

An advantage of this method is that because it refers to a feature of genetic history rather than a phenotype, it need only be done once for any trait under study on the same cohort. While the fitness - phenotype relationship will be different for all traits, that is modeled by the fitted parameter 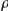 rather than modification of 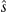. If the impact of LD structure on the prediction does not vary too much between genes, the calculation can be recycled for multiple genes under study. In some experiments, we found the impact of LD to be minimal (data not shown). Discussion of taking the DFE as known versus estimating or using some other flexible function of frequency it is included in Text S1. Discussion of the quality of the existing DFE estimates are also included in Text S1. We have used the observed frequency to estimate the fitness effect, but there are many other potential predictors of functional status. Discussion of including them in our model is found in Text S1.

### Model Fitting and Estimation

#### Testing

Our model fitting procedure will be likelihood-based, so we will use a standard hypothesis testing method: likelihood ratio tests. To improve robustness, our examples will use permutation p-values obtained by comparing the likelihood ratio of the fitted model to that generated under the null hypothesis by randomly swapping genotype vectors between members of the same ethnicity. Permuting genotype labels simulates the null hypothesis that no relationship exists between any genotype and any aspect of the response, which in our parametric setup is equivalent to 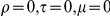 while retaining the relationship between covariates (such as age and sex) and phenotype. Because the genotypes of members of different ethnicities are not exchangeable even under the null, we only swap genotype vectors among individuals with the same reported ethnicity. In admixed populations where information about local ancestry is available, the permutation should be between individuals with the same local ancestry.

#### Estimation

For numerical convenience and statistical robustness, we will use only the first two moments of the model in equations (1), (4), and (6), and assume 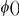 constant in (6). This last restriction yields a mixed-effects regression problem where the genotype effects are crossed random factors, presented in (8) and (9) below. A broad introduction to mixed effects regression and many of the formulas we will use are provided in McCulloch and Searle [37]. In matrix notation where each participant is a row and effects are column vectors,
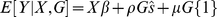
(8)

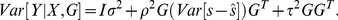
(9)


We allow the procedure to exploit the possibility that individuals with a high burden of rare alleles not only have drift in their mean phenotype because of 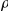 in (8), but also more variability in phenotype due to 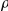 in (9). Equations (8) and (9) assert that a single parameter 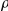 regulates the change in mean variant effect and effect variability with frequency. However, non-differential error (with respect to phenotype) in imputing covariates biases coefficient estimates towards the null, so if our estimations of 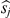 and 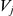 have different levels of error they will experience different such biases. As a result, we will want to fit 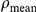 in (8) and 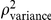 in (9) separately to check that they are similar before combining them. Because it involves an extra parameter the “split 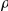
” calculation will be more variable under the null and less powerful when the model is true. However, it may be more robust when the model is mis-specified, as we will explore in our simulations.

We will fit the mixed effects model (8)–(9) using modified Newton-Raphson optimization of the implied likelihood. The linear mixed effects approach is equivalent to assuming normality for the error terms 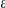 and 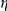 and fitting via maximum likelihood. A major advantage of this estimation approach is that it allows for very fast computation; the likelihood can be integrated analytically over 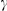 when maximizing over parameters 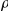 and 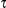. We have not optimized our software for speed, but it completes in a few seconds for the large example dataset. Though higher-order expansions are possible, others have shown that most of the information is often contained in the first two moments of the data [38], [39], and that correct specification of mean and variance models produces correct inference robust to additional details of structure. Assumptions which better match the data at hand will lead to more power, but they will tend to require dramatically more computational effort. For our current example we have considered a single sequenced candidate gene where computational speed is not crucial, but we expect that methods similar to ours will be required for whole-genome or whole-exome resequencing efforts where computational resources will be a limiting factor. Additionally, popular methods such as Markov Chain Monte Carlo and EM which can use arbitrary distributions of residuals and random effects require accurate initial estimates to perform well; MCMC also benefits enormously from a good proposal distribution. Mixed effect regression is a reasonable way to generate these initializations. Whereas using only the first two moments for estimation is only optimal under the normality of 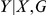, it will still yield consistent estimates if normality does not hold, and we can use use robust methods of testing the null hypothesis such as permutation p-values. This quasi-likelihood-based method also yields best linear unbiased estimates for the SNP phenotype effects [37, chapter 6], which we relied upon in “model interpretation”.

#### Implementation

As discussed above, we will be interested in fitting distinct 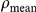 in (8) and 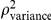 in (9) because of concerns about different magnitudes of error in the computation of 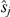 and 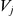. In such a scenario, we can use the SAS mixed procedure [40] to estimate the model parameters and check our custom software. Example code implementing this use is maintained at the authors' website. We generate confidence intervals using the standard asymptotic arguments in McCulloch and Searle [37, chapter 6], which are built into SAS.

Alternatively, if we use a single 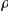 in the mean and variance models, the result is a model which is not easily fit in any standard statistics package of which the authors are aware. We have created a set of functions in the R programing language [41] to estimate this model using optim to maximize the likelihood, code for which is posted at the authors' web site: http://home.uchicago.edu/~crk8e/papersup.html

#### Bayesian interpretation

Our model is easily recast in a purely Bayesian framework. One would need to write priors for 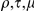 and the effects of covariates. The frequentist formulation is just the Bayesian formulation with an improper uniform prior distribution on the variance components. As a result, using Bayesian regression software like R's MCMCglmm package or winBUGs is an alternative for estimation. A reasonable way to generate proper informative priors would be a three step calculation. First, estimate a posterior distribution on variance explained by genetic factors from previous linkage studies. Because many phenotypes may not have available linkage studies or very low resolution, one may have to rely on other phenotypes or animal model results. Second, equate the resulting prior on attributable variance to the expression in Text S1 with observed values for the genotype data. Third, assign an arbitrary fraction of the explained variation to each source and back-calculate to find the square of the parameter.

The Bayesian analyst could continue to use our normal approximation of the distribution of the latent 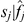 which allows it to be integrated out, or could model it directly including the point mass at zero and skew distribution from the simulation result. The result would be a large model with many latent variables, some of which are poorly identified.

## Results/Discussion

### Dallas Heart Study: ANGPTL4

#### Description of dataset

About 3500 prospectively sampled individuals from the population in Dallas, Texas, were sequenced at a candidate gene for dyslipidemia: ANGPTL4 (Ensembl Acc:16039). These individuals come primarily from three ethnic backgrounds: non-Hispanic white (N = 1043), non-Hispanic black (N = 1832), and Hispanic (N = 601). We will exclude from our analysis the 75 individuals listed as “Other” ethnicity. Our outcome phenotype is log-transformed serum triglyceride levels. Details of the cohort [42], its metabolic phenotypes [43], and the sequencing methods and discovered genetic variation [8], [12] have been described previously. We grouped all missense and nonsense mutations into a single category which we label “non-synonymous” in the tables and figures, and we grouped all synonymous and non-coding region mutations into a single category labeled “non-coding.” Table 1 shows the number of discovered SNPs in each category in each ethnic group. We consider age, sex, ethnicity, diabetes status, and self-reported ethanol consumption as adjuster covariates. For age, we use a flexible linear spline model with knots at every ten years to allow for nonlinearity in response. We include all interactions between ethnicity and gender and ethnicity-gender interactions with other covariates. Because statin use is an endogenous variable indicating diagnosed dyslipidemia, we do not adjust for it. We fit models 1) ignoring statin use and 2) increasing triglyceride levels 25% in the treated to approximate their untreated level. Because we obtained qualitatively similar results, we present only the latter.

**Table 1:** Genetic variation in ANGPTL4.

Population	N individuals	N Non-synonymous variants	N Non-coding variants
Pooled	3476	32	62
Non-Hispanic whites	1043	20	23
Non-Hispanic blacks	1832	15	38
Hispanic	601	8	17

#### Model estimates

Table 2 presents model summaries and point estimates with asymptotic standard errors for model parameters, stratified by ethnicity and pooled using ethnicity as an adjuster. Table 2 presents the results setting the offset term 
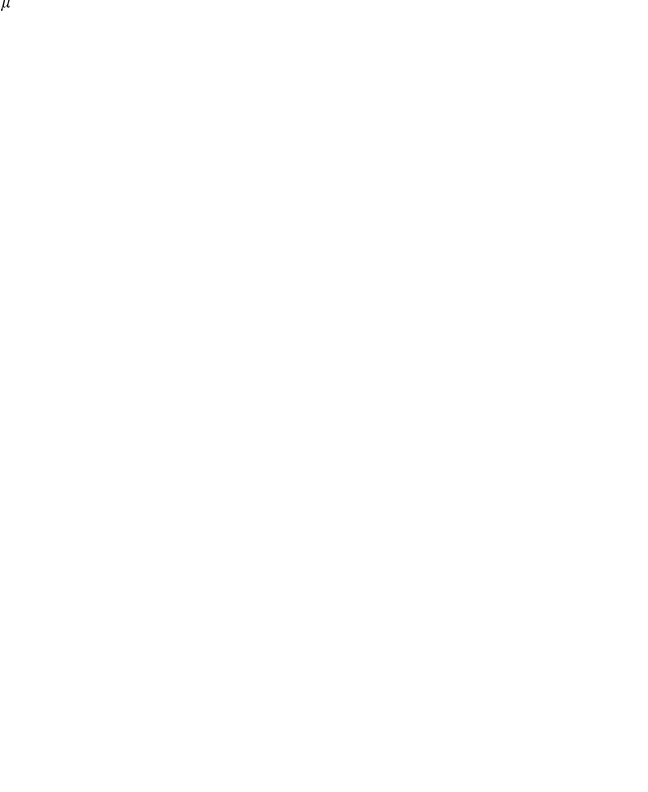
 to zero. We found that including 
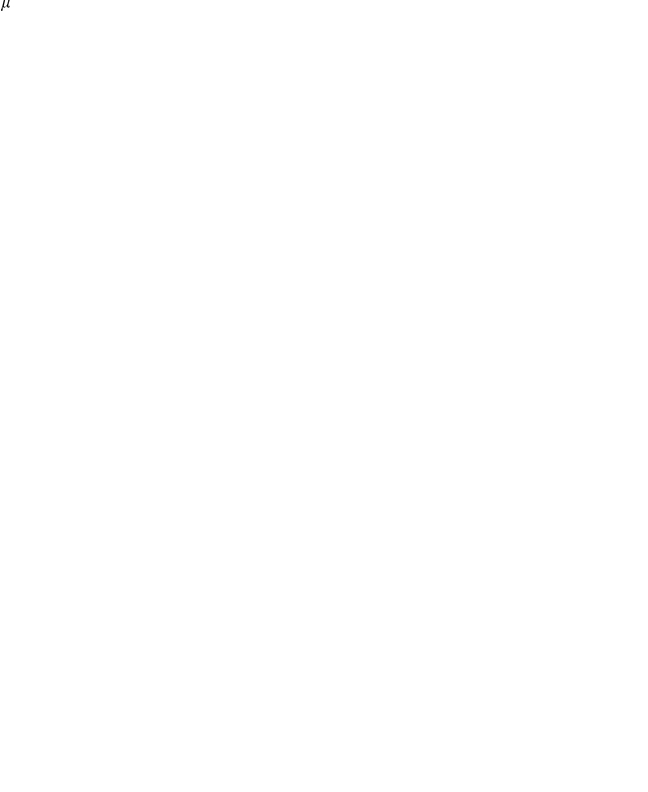
 in (4) produced poor fits when there were few variants, for example when using only the Hispanic non-synonymous variants (n = 8). In the pooled estimate, including the offset did not qualitatively change the result.

**Table 2:** Model fit for ANGPTL4.

Population	SNP Type	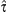	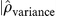	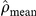	SE	nonfitness % variance	fitness % variance
Pooled	non-syn	0.13	0.0	2.5	8.7	0.54	0.003
Pooled	non-coding	0.02	8.3	−9.6	6.5	0.09	0.08
NHW	non-syn	0.15	0.0	5.8	13.5	0.53	0.03
NHW	non-coding	0.02	0.0	1.9	7.3	0.004	0.008
NHB	non-syn	0.08	0.0	0.5	11.4	0.42	0.0002
NHB	non-coding	0.02	0.0	−11.4	8.1	0.07	0.13
Hispanic	non-syn	0.00	0.0	20.5	43.9	0	0.03
Hispanic	non-coding	0.10	19.6	−40.8	38.2	0.08	0.66

Parameters are defined in equations (1), (4), and (6). SE is for 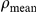. Attributable variance is that due to decomposition (7), see Text S1 for calculation. Pooled model *p* = .0064 on 10000 permutations. Pooled model residual variance = 0.29. NHW is non-Hispanic white; NHB is non-Hispanic black.

For ANGPTL4, we observe a p-value of .006 on 10,000 permutations versus the strong null hypothesis that no SNPs have any effect. Previous authors [12] observed a p-value for a net surplus of non-synonymous variants in low triglyceride participants of .016 and a minimum variant-at-a-time p-value of .019 for E40K corrected for multiple testing. The improvement to the model fit by including 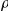 is small in this case; a likelihood-ratio p-value using the asymptotic distribution is non-significant. As seen in Table 2, a glimmer of a fitness component is only seen in the non-coding variation, and the explained variance is very small. However, to illustrate the interpretation of the plots which our approach generates we'll take the parameter estimates at face value below.

#### Interpretation of diagnostic plot

**Figure 3:**
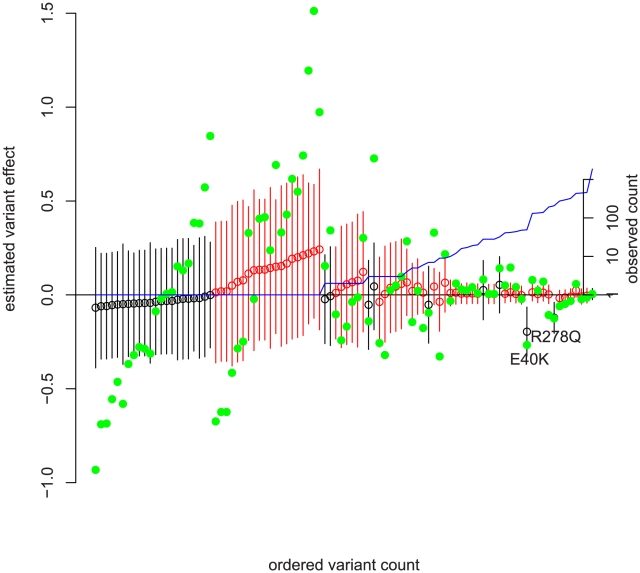
Frequency versus estimated effect size in ANGPTL4 with ordinary least squares estimates. The SNPs have been rank-ordered by observed frequency on the x-axis with ties broken by estimated effect size. The left y-axis is the predicted effect (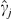 in (4) ) on the log of serum triglycerides. Green solid dots are the point estimate for each variant's effect on log-triglycerides from one variant at a time ordinary least squares adjusted for non-genetic covariates. Open circles are joint point estimates of 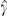 from our method, and bars 95% prediction intervals on those estimates. Confidence intervals are the elementwise Wald-type estimates described in chapter 6 of [37] and produced by SAS's estimate command in the mixed procedure. See Text S1 for the calculation of point estimates, and the sample code at the author's website for SAS commands. Non-synonymous variation is in black; non-coding variation in red. The right y-axis and blue line depict observed count pooled across ethnicities on a log scale.

Figure 3 shows the observed SNPs and estimated effect sizes (non-synonymous in black and non-coding in red) rank ordered by observed frequency (in blue). Variant-at-a-time ordinary least squares (OLS) estimates of effect size are overlaid in green. Figure 3 displays several interesting features of the data; first there are two low-frequency non-synonymous variants with a strong effect reducing triglyceride levels; the first is E40K (frequency in non-Hispanic whites = .012, frequency in non-Hispanic blacks = .003), the sole variant identified by Romeo et al [12]. However, adjusted for E40K we see that another more common variant R278Q almost exclusive to non-Hispanic blacks (frequency = .055) also appears to decrease triglyceride levels. We observe a weak tendency for all non-synonymous variation to reduce the phenotype; Romeo [12] also noted an excess of rare non-synonymous variants in those with low triglyceride levels. The rare non-coding variation appears to have the opposite sign of effect; it increases triglyceride levels. Referring to Table 2 we see that a fitness-related component of variability (of about the same scale as the change in mean) was detected; this gives rise to the wider spread of point estimates and wider confidence intervals in non-coding variation.

An interesting data point in Figure 3 is a single 5% frequency non-coding variant (directly before R278Q) whose OLS effect estimate is quite large (and nominally significant) but whose model-based effect estimate is small. Examining that variant more closely, we found that it is in strong LD with R278Q. Because E40K (which is not strongly correlated to any other variation) had a large effect and non-synonymous variants tended to decrease triglycerides, the model assigned non-synonymous variation as more likely to have non-rare variation with large negative effect sizes and gives the effect to R278Q. Similarly, perfectly correlated rare variants have their combined effect split evenly.

We can understand this model fit by looking at the green OLS estimates in Figure 3. Visually, the estimates for non-synonymous variation tend to be below zero. Comparing the non-synonymous to non-coding singletons, we see more variable estimates in the non-coding singletons as well as a different mean. The model fit identifies this as opposite signs of 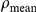 and a much greater 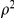 in non-coding. The non-rare non-synonymous variants with large effects (E40K, R278Q) drive the larger estimate of 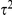 versus non-coding variants; examining Figure 2 we see that common variation is essentially neutral with respect to fitness, and as a result non-zero effects in non-rare variants force 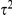 away from zero.

#### Evolutionary interpretation

An interesting potential story about natural selection on ANGPTL4 activity emerges from Figure 3. First, non-synonymous mutation tended to decrease the effectiveness of ANGPTL4 and decrease serum triglyceride levels [8], [44], [45]. We see no evidence of selection against those mutations; variants which decreased triglycerides became more than rare in both the African and European lineages, and we see no excess of large effects in rare SNPs. On the other hand, non-coding mutations which may alter the regulation of ANGPTL4 on average increased triglycerides. Variants with large effect sizes were preferentially rare, and the apparent selective force was stronger in the non-European lineage, as the demographic history would predict. This meshes well with the finding that ANGPTL4 experienced a Europe specific relaxation of purifying selection [12]. We do not suggest that serum triglyceride levels in themselves were the target of purifying selection; effect on triglycerides may only be correlated to effect on a selected function.

### Simulation Studies

#### Population parameters

In order to determine the power and robustness of our procedure, we simulated variation in a gene with the exon structure of the gene ANGPTL4 in a study population using SFS_CODE [36] and fitted demographic and DFE parameters [31], [33]. We used 4cM/mb for the local recombination rate and no recombination hotspots. We used 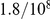 as the mutation rate per-nucleotide-per-generation. From the final simulated population of about 20,000 individuals we sampled 1000 individuals independently for each of 1000 simulation runs. SFS_CODE commands creating the simulated population are available at the authors' web site. We created simulated phenotypes according to (1) and (2) using parameters described below. The total simulated population had 132 coding-region SNPs, 29 of which were at frequency greater than 1%.

#### Model parameters

We chose several levels of the phenotype parameters to correspond to potential cases of interest while keeping the total fraction of variation explained by the gene about the same: a weak mean variant effect, a strong fitness-related component of the phenotype, and a strong fitness independent component of the phenotype. We chose the baseline values such that 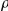 and 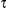 explain about the same amount of variation in phenotype. We also created a scenario with no fitness-phenotype correlation whatsoever. To ensure that type 1 error rates were correct, we include a simulation under the null hypothesis that no variants have any effect on phenotype. Table 3 contains the chosen phenotype parameter values for each set of simulations and the resulting expected percent of variance explained by the SNPs due to fitness-phenotype correlation and percent of variance explained independent of that correlation.

**Table 3:** Simulation design.

scenario	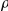	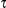	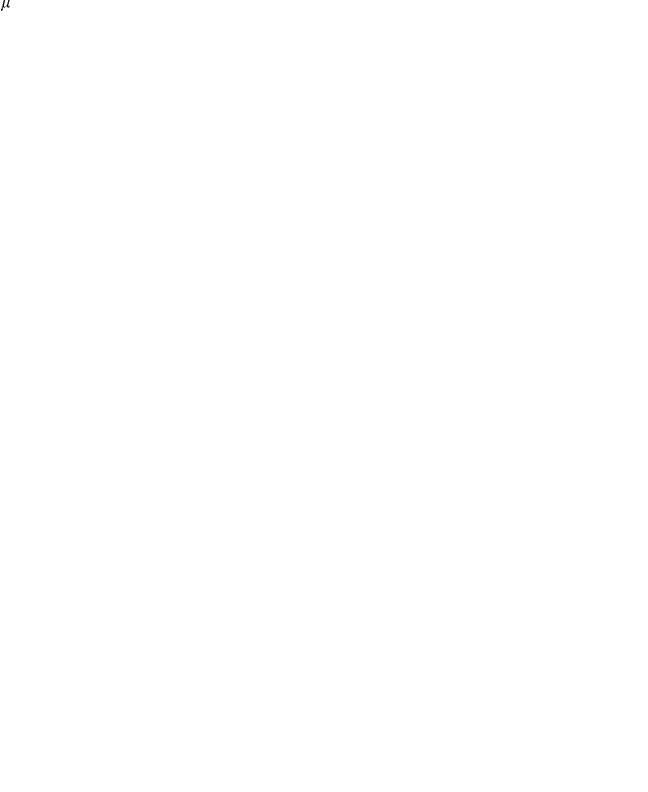	residual standard deviation	expected fitness % variance explained	expected nonfitness % variance explained
Base	−7.0	0.012	0.007	0.22	0.84	0.84
High 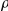	−21.0	0.012	0.007	0.50	1.51	0.17
High 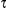	−7.0	0.018	0.007	0.28	0.55	1.13
Low 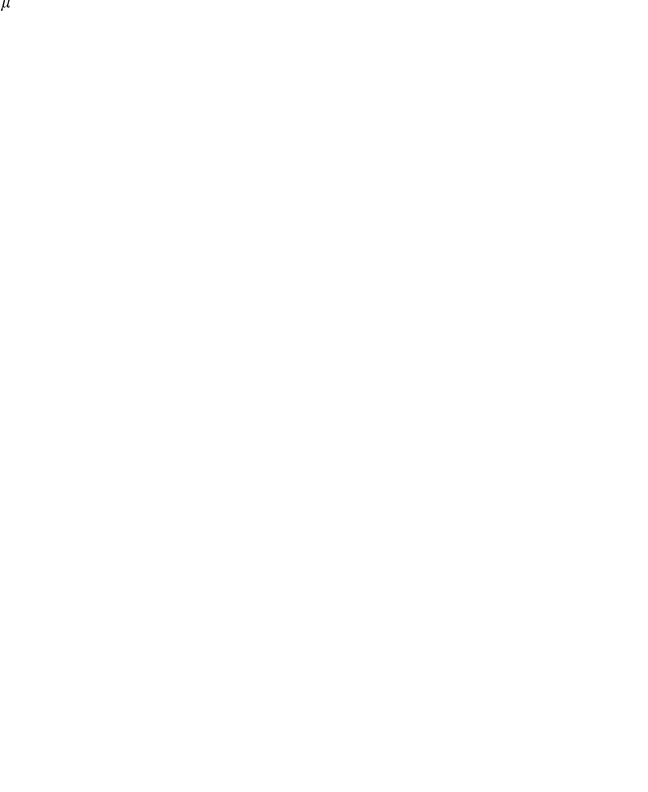	−6.4	0.012	0.003	0.21	0.83	0.85
Very high 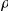	−63.1	0.012	0.003	1.43	1.66	0.02
Zero 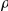	0.0	0.012	0.007	0.16	0.00	1.68

Parameters chosen for simulation. Data generated by mechanism of formula (1) and (2). Parameters defined in equations (1), (2), and (6). Explained variance is the average true variance over individuals of fitness component and fitness independent component.

Two additional batches of simulation examine the robustness of our procedure to incorrect assumptions. First we created violations of the assumed population model. We mis-specified the assumed DFE in our analysis, making the scale parameter a factor of 5 too large or too small and keeping the truth the same. We also simulated violation of our demographic assumptions using a population which experienced an additional 100 fold exponential growth over the last 11% of generations since out-of-Africa. Second we created violations of the assumed statistical model. We simulated three scenarios violating the linearity assumption. First, with 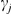 proportional to 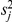, second proportional to 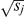, and third a 50/50 mixture of 
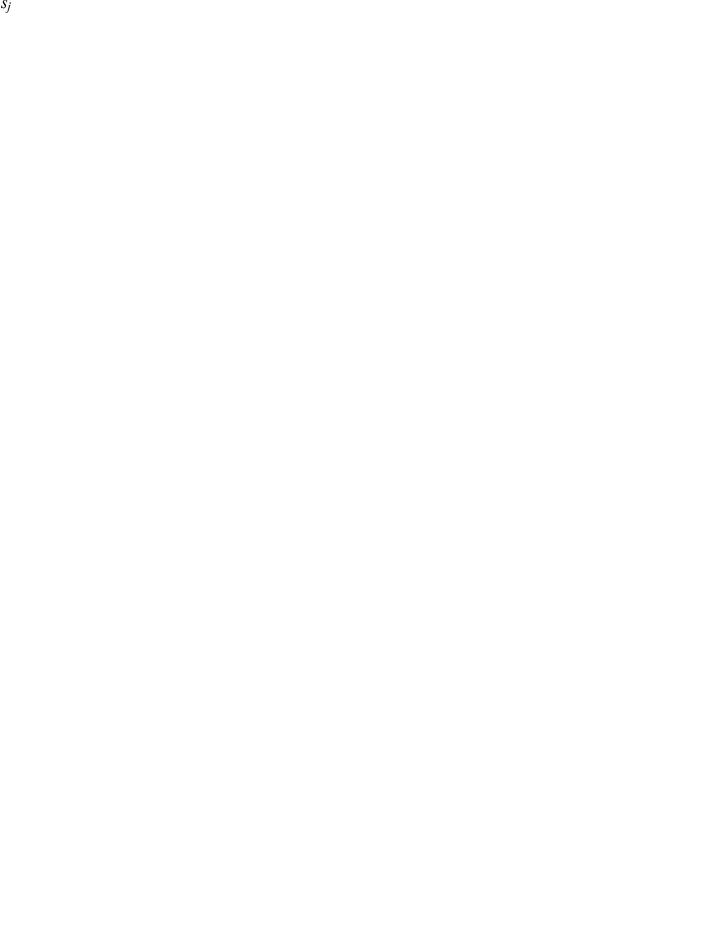
 and 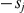. We simulated 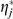 using a highly skewed log-normal distribution which was then standardized to have mean zero and variance 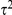. We also simulated with 20% and 80% of the variants having an effect size of zero.

#### Power comparisons

To compare power with existing methods, we included several proposed methods of analysis. First, we test the method of Bonferroni corrected minimum p-value of SNPs with minor allele frequency >1% or >5%. Other proposed methods using allele counts like CAST [46] CMC [20] and weighted sums [21] were created for case-control studies, so we alter those methods to be fair in a cohort quantitative-trait context. Our representative of CAST-like analysis is regression with the number of rare variants carried by each participant as a covariate; CMC-like analysis is the same with non-rare SNPs (frequency greater then 1% or 5%) treated as free regression parameters. P-values are then generated by ANOVA against the nested model consisting only of only fitting the mean response. Our representative of weighted-sum type methods is a similar regression analysis where rare variants are collapsed to a mean model with burden proportional to 
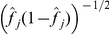
, which is the same weight used by Madsen et al [21]. Because the simulated response is actually normal, we do not use a rank transformation. We also used the same burden function for only low frequency SNPs and treated common SNPs as regression parameters. P-values are again obtained by ANOVA versus a nested model with no genetic effects.

To demonstrate the gain (or loss) in information by considering the marginal variance, we apply a similar regression with an optimal mean model, that is (8) either for all SNPs or treating common SNPs as free. We tested our model both with a single 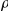 in the mean and variance and the “split 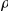
” calculation where separate parameters are fit in (8) and (9).

Table 4 summarizes the power comparisons in each case. Our model is as or more powerful than the existing methods, even when there is substantial violation of its assumptions. The only scenario in which our model loses some power is when there is absolutely no fitness-phenotype correlation. Even in that case, the relative loss is small, much smaller than the gain when 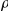 was not zero. The additional utility of the method varies substantially depending on the chosen parameters. For example, when the fitness-phenotype correlation accounts for about half the genetic component of the phenotype (the basic scenario), our method provides a substantial improvement, but when 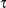 is large (common variants have large effect sizes) the benefit is less. Our model appears reasonably robust to all the violations of assumptions which we tested, even providing a performance benefit when effect sizes were very skew or the true relationship was nonlinear. In effect, the truth in those cases lined up less well with the implicit assumptions of the competing methods. Perhaps most importantly, even fairly substantial mistakes in the DFE and demographic history did not dramatically reduce the power of our method. The “split 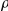
” model appears to perform about the same as a single 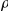. The minimum p-value method's poor showing in some scenarios is explained by the data generating mechanism we chose; when 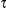 is small or many SNPs have zero effect there will often be no common variants with appreciable effect sizes.

**Table 4:** Simulation study power results.

	Min *p*		CAST		CMC		Weighted Sum		Optimal Mean		EMMPAT	
scenario	1%	5%	1%	5%	1%	5%	All	5%	All	5%	Split 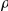	One 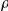
Null	.05	.05	.05	.05	.04	.06	.05	.04	.04	.05	.05	.04
Base	.22	.26	.30	.27	.30	.36	.22	.35	.45	.39	.54	.56
High 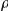	.06	.08	.12	.11	.08	.10	.26	.13	.38	.21	.48	.48
High 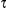	.28	.34	.27	.27	.42	.46	.18	.45	.36	.47	.52	.53
Low 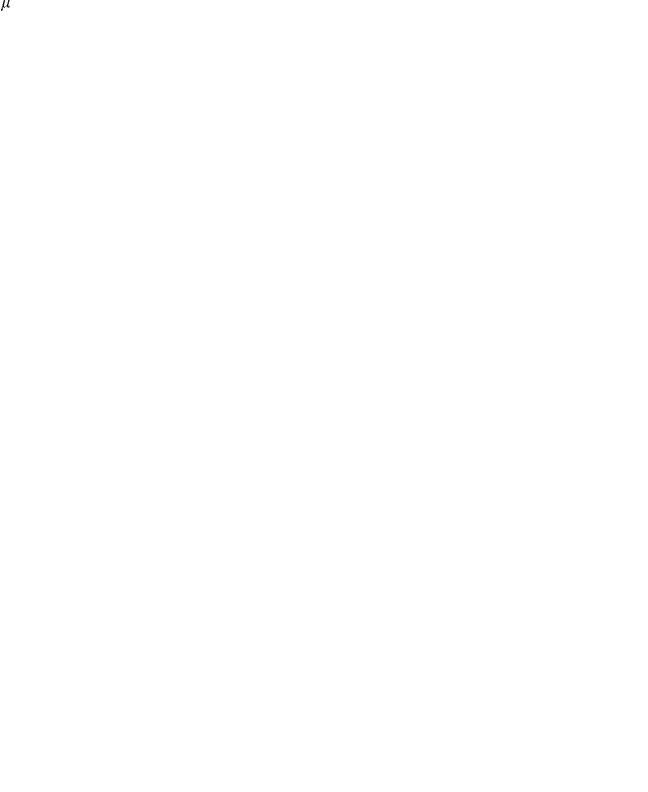	.20	.26	.21	.21	.32	.36	.18	.38	.36	.42	.49	.48
Very High 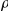	.04	.06	.06	.06	.05	.06	.22	.08	.32	.15	.45	.45
Zero 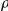	.44	.52	.48	.44	.61	.66	.09	.62	.45	.61	.58	.62
	violation of population model assumptions											
DFE*5	.23	.28	.30	.27	.33	.39	.24	.39	.43	.42	.51	.53
DFE*.2	.22	.27	.33	.30	.33	.36	.24	.37	.46	.39	.56	.55
Exponential growth	.29	.32	.37	.35	.46	.51	.13	.49	.38	.48	.50	.53
	violation of fitness linearity and distribution of 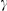 assumptions											
Square	.20	.23	.28	.23	.29	.33	.12	.31	.39	.39	.57	.59
Random sign	.20	.27	.28	.25	.32	.36	.10	.33	.28	.34	.43	.45
Square root	.23	.27	.35	.28	.35	.34	.23	.39	.43	.41	.49	.48
Skew effects	.22	.29	.34	.31	.33	.37	.24	.38	.45	.41	.56	.56
20% no effect	.30	.33	.31	.28	.40	.45	.24	.46	.45	.50	.59	.60
80% no effect	.33	.33	.18	.17	.35	.36	.13	.36	.24	.38	.44	.43

Simulation parameters are described in Table 3. 1000 replicates were generated for each scenario. Assumption violating scenarios are described in the text. Simulations on violation of model assumptions use the same parameters as “base”. Where nonlinear transformations of fitness effects are used, the variance of transformed fitness effects is rescaled to be the same. Power is proportion of *p* less than .05. Min *p* is Bonferroni corrected minimum p-value; CMC is the method of [20]; CAST is the same method ignoring common variants; Weighted is the method of [21]. Optimal Mean uses our implementation of 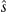, but not variance components. See methods text “Simulation Studies” for how CAST, CMC, and Weighted were modified to be closer to the data generating mechanism. Columns with “1%” or “5%” involve dichotomizing variants at the specified frequency threshold. CMC, Weighted, and Optimal Mean treat variants above that threshold as free regression parameters. Our method (EMMPAT) likelihood ratio *p* value is estimated from 500 permutations.

### Discussion

We propose a novel method, EMMPAT, for association between sequenced genes and phenotype which utilizes population genetic theory to pool information among rare variants. Our method generalizes allele-count and allele-burden techniques, and presents several advantages. Of greatest importance to the practicing scientist will be increased power and interpretability. As shown above, our method allows us to leverage allele frequency as auxiliary data related to SNP effects and to substantially increase power to detect association in many scenarios. The availability of a well motivated pooling strategy allows an omnibus test which incorporates common and rare variation simultaneously. Our approach provides clear interpretations for the fitted model, such as the attributable variance in phenotype due to all polymorphisms observed in a gene, particular types of SNPs, or only the rare variation. Furthermore it facilitates tests of meaningful parameters (such as mean derived allele burden) and group differences (such as non-synonymous versus non-coding). The regression toolbox allows model checking and exploration, such as in Figure 3 which presents the data in an informative format. Additional model checking proceeds as usual in linear mixed models, and posterior predictive checks are similarly possible.

A relevant question is how important our method will be for diseases which have not been strongly selected against. There are three answers to consider. First, when selection and disease effect are completely independent, common SNPs will tend to have just as large effect sizes as rare SNPs and explain much of the heritable variation in phenotype [2], [3]. We believe that most investigators conducting resequencing studies assume rare variation to have larger effect sizes, since that is the best-justified scenario for the expense of sequencing. Second, our method allows for this possibility in the form of estimating 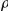 to be zero and 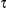 non-zero. As demonstrated in our simulations, the loss of power in adding a single unnecessary parameter to describe many SNPs is small. Third, as discussed in the Introduction and Text S1, direct selection against disease is not a necessary condition for correlation between fitness and phenotype; as long as the disease related gene is under selective pressure in any of its functions, we expect a correlation.

We have planned several extensions to this method. In addition to improved techniques of estimating fitness effects, we need to incorporate evidence for adaptive selection. Signatures of positive selection [47]–[49] can be used to prioritize genes for study which may have been more important in differentiating humans from our ancestors and hence contribute to modern phenotypes. We expect positively selected variants to have very different phenotype effects from neutral alleles, but it is not clear a-priori what that relationship should be or if it will be possible to reliably identify positively selected SNPs [50], [51]. Second, for mathematical and numerical convenience we have developed this method in the context of a prospective probability sample measuring a quantitative trait. Both these assumptions need to be relaxed for the setting of most resequencing projects. Disease phenotypes are frequently non-normal, binary, or censored such as time-to-event from clinical trials, requiring a generalized linear mixed model. The prospective sampling assumption will also require work to relax. Retrospective sampling such as in case-control designs and extreme-phenotype-based sampling [13], [52] is well known to distort random effect distributions [53]. Third, in our example and simulations, we assume that 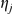 are independent of one another, but one need not do this. One could add spatial covariance structures between 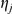 to relax the independence assumption, which would correspond to allowing that variants nearby each other in the genome or folded protein tend to have similar effects. Especially in exome-only resequencing studies, consideration of unobserved linked markers with techniques similar to TreeLD [35] will be important. Our model has not included dominance or epistasis between SNPs or genes, the structure of which is probably not simple, although progress has been made on determining the impact of these features to quantitative traits [54], [55]. Finally, because our example dataset comes from high-quality Sanger sequencing, we have ignored nonrandom missing data issues. Future work involving second generation sequencing or beyond must address the complex nature of library coverage, alignment error, and genotyping error inherent in those technologies.

## Supporting Information

Text S1 Supplementary methods and discussion.
(0.05 MB PDF)
