# Supplementary material for: An Evolutionary Framework for Association Testing in Resequencing Studies
Source: PLoS Genet. 2010 Nov 11;6(11):e1001202. doi: 10.1371/journal.pgen.1001202 (PMC2978703; doi:10.1371/journal.pgen.1001202)
Supplement: Text S1 — Supplementary methods and discussion. (0.05 MB PDF) [file pgen.1001202.s001.pdf]

Supplementary Text

0.1 Relationship to Previous Methods in the Evolution of Quantitative Traits

The main text uses a simplified development based on fairly strong assumptions: directional selection, and independent pleiotropic effects. Several alternatives are available; these alternatives also illustrate the potential violation of the linearity assumption. Previous work which describes the evolution of quantitative traits starts with phenotype effects and derives conditional fitness effects [1]. For example, using the stabilizing selection approximation of a gaussian penalty for square effect size (nor-optimal approximation):

$$s_j = a\gamma_j^2. \quad (1)$$

The square penalty has theoretical basis (suitably differentiable functions are locally quadratic around their optima), but is largely chosen for mathematical tractability. Under this assumption, conditioning on observed frequency yields

$$E[s_j|\hat{f}_j] = a(E\left[\gamma_j|\hat{f}_j\right]^2 + Var\left[\gamma_j|\hat{f}_j\right]) \quad (2)$$

and

$$E\left[\gamma_j|\hat{f}_j\right] = \sqrt{1/a}E\left[\sqrt{s_j}\frac{LR(\pm\sqrt{s_j})-1}{LR(\pm\sqrt{s_j})+1}|\hat{f}_j\right] \quad (3)$$

where $LR(\pm\sqrt{s_j})$ is the likelihood ratio for γ_j equal to positive and minus $\sqrt{s_j}$ using the current parameters of the distribution of γ. This scheme can be plugged into our framework using the simulated distribution of $s_j|\hat{f}_j$, albeit with somewhat more difficulty in optimization. Of note, if γ are all the same sign for a particular gene under polygenic stabilizing selection, then this reduces to

$$E\left[\gamma_j|\hat{f}_j\right] = \sqrt{1/a}E\left[\sqrt{s_j}|\hat{f}_j\right] \quad (4)$$

$$Var\left[\gamma_j|\hat{f}_j\right] = 1/aE\left[s_j|\hat{f}_j\right] - 1/aE\left[\sqrt{s_j}|\hat{f}_j\right]^2. \quad (5)$$

This model is quite similar to that proposed in the main paper; differentiating between them will require empirical testing on large datasets.

Loosening the assumption to a stabilizing selection with pleiotropic effects of unknown joint distribu-

tion with phenotype effects and only specifying conditional moments is also possible. Something similar to our model arises by giving s "at birth" the form of a correlated pleiotropic effect

$$s_j = \gamma_j^2 u + z, \tag{6}$$

with u independent of z and γ, that is, a random piece of the phenotype effect becomes a fitness effect along with a random impact not mediated by the phenotype. While it seems straightforward to proceed as above with iterated conditional expectations of s_j and a Taylor expansion of $E\sqrt{s_j}$, conditioning on the observed frequency removes the independence and complicates the development. Going further will require either making additional assumptions or use of more advanced semiparametric techniques, which we leave for future work. Special attention must be paid to the boundary conditions around neutrality [1], as further discussed in the section on attributable variance, as well as ensuring compatible marginal and conditional distributions. Our current model requires that selection acts through pure pleiotropy since we do not require neutral alleles to have zero effect.

The complete generality of allowing arbitrary joint distributions of pleiotropic effects and phenotype effects or arbitrary nonlinear forms of stabilizing selection comes with an intractable analytical problem. Appealing to methods based on the first few moments of the joint distribution, as we do, is one away around that problem. Additionally, since we do not have an idea of what the marginal distribution of γ for new mutations should be a-priori, modeling its moments conditional on s, for which we do have a marginal distribution, is easier. Semiparametric methods or using the empirical distribution of $\hat{\gamma}$ are another way forward.

0.2 SNP Effect Prediction and Attributable Variance Calculations

The calculation of the variance explained by genetic factors can proceed in two ways. First, we can follow the derivation of Johnson and Barton [1] (their appendix A) which requires that $E\left[\frac{\gamma^2}{s}\right]$ be finite. The formula there yields in the diffusion approximation

$$V_G \propto E\left[\frac{\gamma^2}{s}\right] \tag{7}$$

$$\propto \rho^2 E|s| + \tau^2 E s^{-1}. \tag{8}$$

However, the second term is infinite in our chosen DFE because it contains a point mass at zero; the positive variance of phenotype effects among these neutral mutations creates infinite $E\left[\frac{\gamma^2}{s}\right]$. Were we to substitute a DFE with finite harmonic mean, this would provide a separation into a selection dependent first term and selection independent second term. To avoid the reliance on the diffusion approximation, we could in the same spirit write the total variance using an iterated conditional expectation

$$V_G = Var\left(G_i\gamma\right) \tag{9}$$

$$= E\left[Var(G_i\gamma|G_i)\right] + Var\left[E(G_i\gamma|G_i)\right] \tag{10}$$

$$= E\left[\sum_j G_{ij}^2\right]\tau^2 + E\left[\sum_j V_j G_{ij}^2\right]\rho^2 + Var\left[\sum_j s_j G_{ij}\right]\rho^2 + Var\left[\sum_j G_{ij}\right]\mu^2 \tag{11}$$

where the outer variances and expectations are across individuals in the sample. The interpretation of these estimates is unintuitive; they describe an average variation explained over *possible sets of SNPs* in a new population with identical SNP structure to the present study. That may not be interesting to practicing epidemiologists who want to know about the variance due to SNPs which actually exist.

We can also try to build attributable variance based on predicted effect sizes. A formula for best linear unbiased prediction of random effects is provided by McCulloch and Searle [2, chapter 6]. Define V^- as the matrix inverse of $Var(Y|G, X, \rho^2, \tau^2, \sigma^2)$, and the matrix $P = \left(V^- - V^- X\left(X^T V^- X\right)^- X^T V^-\right)$ then we can estimate the total SNP effect contribution to each person

$$G\hat{\gamma} = \rho G\hat{s} + \mu G\{1\} + G\,Var(\gamma)G^T PY \tag{12}$$

$$= \left[\tau^2 G G^T PY + \mu G\{1\}\right] + \left[\rho^2 G\,Var\left[s - \hat{s}\right] G^T PY + \rho G\hat{s}\right] \tag{13}$$

which are fitness independent and fitness dependent components. Taking the sample variance over subjects of each half of SNP effect contribution then produces attributable variance. This formula relies on getting effect sizes right, and may be more sensitive to incorrect specification. Because the model ignored features like the higher moments of the DFE and point mass at zero, a certain amount of mis-specification is guaranteed. Additionally, because of the phenomenon of shrinkage whereby random effect estimates are biased towards zero, the above estimates will tend to systematically underestimate attributable variance. In particular, while the model allows that, as a collection, the effects of rare SNPs are systematically larger than common SNPs, because there is little information per-rare-SNP the effect estimates are strongly

shrunk to a common value.

Formulas for the asymptotic sampling variance of random effect estimates are also provided in [2], and these are used to generate confidence intervals. These asymptotics are known to converge somewhat slowly, so the coverage properties may not be exact.

0.3 DFE as known versus estimating its parameters

We take the DFE as known in our procedures, but for investigators with large datasets it may be tempting to re-estimate its parameters, perhaps on a per-gene basis, to calibrate our assumed relationship between observed frequency $\hat{f}_j$ and mean fitness $\hat{s}_j$. The `prfreq` software [3] uses the observed site-frequency spectra of derived alleles as data to fit an assumed shape of a DFE with an EM algorithm. Because it relies on having many variants to form a reliable empirical distribution of allele frequencies, this method is suitable to collections of genes or genes which contain a large number of variants believed to be suitable for pooling. Similarly, `mkprf` [4] compares the rate of polymorphism and fixed differences across specified genomic areas to estimate selection intensity and rank genes based on evidence for selection. Because more data is gathered by expanding the phylogenetic tree examined for fixed differences rather than by including a broader genomic area, it can estimate the mean fitness effect very locally and is suitable to single genes or even smaller regions.

We do not use gene-specific estimates of the DFE in our approach because neither method implemented in existing software is adapted to our setting. `prfreq` relies on having a large number of variants and is computationally workable only with small sample sizes, but our example has over 3500 participants sequenced on only 3 genes. `mkprf` assumes a hierarchical structure on fitness-effects and requires a larger number of genes to create effective contrast between genes. It also is very sensitive to prior parameters [5]. Because it fits a single fitness effect per-gene, it is not clear how it functions in the presence of both adaptive and purifying selection. Future sequencing studies may better meet the requirements of the above software, and some investigators are attempting to combine both site frequency spectrum and phylogenetic approaches into a single estimate or include more information, such as the apparent age of variants.

One potential problem is that we have taken parameters estimated with uncertainly and treated them as knows. Particularly, estimating demographic parameters from the datasets used in [3] may not be reliable because the small number of people sequenced constrains the extent to which the SFS can inform

on recent demographic patterns. These estimates are doubtless incorrect in some way, and we will lose efficiency as a result. We do not believe our procedure for computing fitness effects to be optimal, but an advantage of our formulation is that as improved methods for estimating of fitness effects become available they can be easily substituted into the association procedure.

Instead of our fairly complicated mechanism for estimating fitness effects, one could instead choose a-priori to have the mean and variance of γ come from a specified form and maximize over its parameters. For example, one could impose that $\hat{s}_j|\hat{f}_j$ followed a power-law and maximize the implied likelihood over its parameter. We found a power law to be a reasonable approximation to $\hat{s}$ in some settings, but not others (data not shown). Such an approach would come with additional flexibility at the price of more variable null hypothesis behavior and hence a loss in power. Because the other parameters in the model could change value dramatically depending on the chosen parameters for $\hat{s}_j|\hat{f}_j$, confidence intervals for them might be adversely effected. Of course should the underlying conditional expectation of fitness not follow the assumed form, we would expect this to be less accurate. It would also eliminate the fitness-phenotype correlation interpretation of the fitted model. Because the population-genetic approach describes the underlying data-generating mechanism for a resequencing study, we prefer it to ad-hoc methods.

0.4 Non-frequency Predictors of Fitness or Function

It would be advantageous to be able to include prior information on SNP effects besides frequency. For example, predictions based on substitution type and sequence composition for non-synonymous variants [6–15] or eQTL maps for non-coding variation [16] would add valuable prior information. Our procedure can be generalized to include them in three ways. First, for those bioinformatic tools already calibrated to predict fitness effect, the features of deleterious sequences which they rely on could be added to the generative model for fitness effects of new mutations in the simulation method. The mean and variance of true fitness effects conditional on observed features of the variants in the real data could then be calculated in the same way as before. Second, for any of the tools which are not calibrated on fitness, their estimates of SNP function can be included as a fixed effect in the model analogous to $\hat{s}$. If the mean prediction error of the tool relative to its underlying construct is available, that prediction error can be included as a variance component analogous to the role of V_j. Third, tools which identify regions as probably relevant (like highly conserved regions) could warrant either stratified parameter estimates

or a correlation between the effects of variants in the region.

References

1. Johnson T, Barton N (2005) Theoretical models of selection and mutation on quantitative traits. Philosophical Transactions of the Royal Society B: Biological Sciences 360: 1411–1425.

2. McCulloch CE, Searle SR (2000) Generalized, Linear, and Mixed Models. Hoboken, NJ, USA: John Wiley & Sons, Inc.

3. Boyko AR, Williamson SH, Indap AR, Degenhardt JD, Hernandez RD, et al. (2008) Assessing the evolutionary impact of amino acid mutations in the human genome. PLoS Genetics 4: e1000083.

4. Torgerson DG, Boyko AR, Hernandez RD, Indap A, Hu X, et al. (2009) Evolutionary processes acting on candidate cis-Regulatory regions in humans inferred from patterns of polymorphism and divergence. PLoS Genetics 5: e1000592.

5. Li YF, Costello JC, Holloway AK, Hahn MW (2008) "Reverse ecology" and the power of population genomics. Evolution; International Journal of Organic Evolution 62: 2984–2994.

6. Frdric MY, Lalande M, Boileau C, Hamroun D, Claustres M, et al. (2009) UMD-predictor, a new prediction tool for nucleotide substitution pathogenicity-application to four genes: Fbn1, fbn2, tgfbr1, and tgfbr2. Human Mutation 30: 952–959.

7. Corbo RM, Prvost M, Raussens V, Gambina G, Moretto G, et al. (2006) Structural and phylogenetic approaches to assess the significance of human apolipoprotein e variation. Molecular Genetics and Metabolism 89: 261–269.

8. Wang K, Horst JA, Cheng G, Nickle DC, Samudrala R (2008) Protein Meta-Functional signatures from combining sequence, structure, evolution, and amino acid property information. PLoS Comput Biol 4: e1000181.

9. Tian J, Wu N, Guo X, Guo J, Zhang J, et al. (2007) Predicting the phenotypic effects of non-synonymous single nucleotide polymorphisms based on support vector machines. BMC Bioinformatics 8: 450.

10. Kumar P, Henikoff S, Ng PC (2009) Predicting the effects of coding non-synonymous variants on protein function using the SIFT algorithm. Nat Protocols 4: 1073–1081.

11. Lee T, Lee A, Li K (2008) Incorporating the amino acid properties to predict the significance of missense mutations. Amino Acids 35: 615–626.

12. Bahadur KCD, Livesay DR (2008) Improving position-specific predictions of protein functional sites using phylogenetic motifs. Bioinformatics 24: 2308–2316.

13. Cheng G, Qian B, Samudrala R, Baker D (2005) Improvement in protein functional site prediction by distinguishing structural and functional constraints on protein family evolution using computational design. Nucleic Acids Research 33: 5861–5867.

14. Ramensky V, Bork P, Sunyaev S (2002) Human non-synonymous SNPs: server and survey. Nucleic Acids Research 30: 3894–3900.

15. Rooman MJ, Kocher JPA, Wodak SJ (1992) Extracting information on folding from the amino acid sequence: Accurate predictions for protein regions with preferred conformation in the absence of tertiary interactions. Biochemistry 31: 10226–10238.

16. Pickrell JK, Marioni JC, Pai AA, Degner JF, Engelhardt BE, et al. (2010) Understanding mechanisms underlying human gene expression variation with RNA sequencing. Nature 464: 768–772.
